# On Gonorrhœa in Its Constitutional Aspects*Prepared to be read at the British Homœopathic Congress, held at Birmingham, September, 1888, and published in the *Monthly Homœopathic Review*, London.

**Published:** 1889-06

**Authors:** J. Compton Burnett

**Affiliations:** London


### HOMŒOPATHIC PHYSICIAN,

A MONTHLY JOURNAL OF

HOMŒOPATHIC MATERIA MEDICA AND CLINICAL MEDICINE.

"If our school ever give up the strict inductive method of Hahnemann, we are lost, and deserve only to be mentioned as a caricature in the history of medicine."—CONSTANTINE HERING.

Vol. IX. JUNE, 1889. No. 6.

### ON GONORRHŒA IN ITS CONSTITUTIONAL ASPECTS; WITH SPECIAL REFERENCE TO THE SYCOSIS OF HAHNEMANN.*

J. COMPTON BURNETT, M. D., LONDON.

For years past I have thought that it would be a very desirable task to be undertaken, to investigate afresh those diseases that give the groundwork of the biopathology of the Seer of Coethen, and I have often wondered that the vigor and enterprise of some of our number of this generation have so long left this field of research comparatively untilled—that is, untilled in this generation. For, in our gropings after truth, each succeeding generation gains a little on its predecessor, by the general progress of knowledge, and by the slow movings of the human mind toward as much of certainty and of finality as seems attainable for the limited and finite.

And then, whether we believe in psora, syphilis, and sycosis or not—that is, as they are taught by Hahnemann—a large part of the work done by the homœopathic school during the past fifty years is more or less tinged with these doctrines; and, moreover, anything taught by so able an observer as was Hahnemann deserves serious investigation at our hands. And,

* Prepared to be read at the British Homœopathic Congress, held at Birmingham, September, 1888, and published in the *Monthly Homœopathic Review*, London.

whatever may be said of the therapeutics of general medicine, positive diagnostics has distinctly advanced during the past decade, and I submit that it is desirable that our own position should be reviewed in the light of this advance.

When I had given the First Hahnemannian Lecture, known as "*Ecce Medicus*," I certainly thought one of my followers in the orator's chair would have tackled the Coethen phase of Homœopathy, and exhibited it in the light of modern research and experience, so as to determine for us of this generation how much of it still holds good, and what part, if any, must be considered as no longer tenable. But, thus far, the work has not been done since then, and I, therefore, will proceed to consider the subject in part here.

Mr. Punch is a great authority for us in this country of spleen and gravity, and, as we all know, his reiterated advice in regard to things to be done is that if you want them done well, do them yourself.

Hahnemann, as is well known, spent his younger and more vigorous days in demolishing theories and hypotheses; indeed, he threw them all right out of his mental window, and made a fresh start altogether with medicine sans pathology, sans theories, sans everything, in fact, but the therapeutic law of similars, which is still for many a very filmy theory indeed. However, the law of likes is no more theory for us; for us it is the one thing common to our body; outside of the law we practically agree about nothing, and yet, notwithstanding this almost general disagreement amongst us, our friends, the enemy, will have it that we and the medical profession at large are not *solidaire;* surely the fact that we disagree about almost everything that is of vital importance should offer them sufficient internal evidence of their and our solidarity.

But, as I said, we agree on our fundamental law, except, indeed, that some of our number of late years have had sad searchings of heart about the law also! It is a rule, they say, not a law! Or, again, it is a method. So that, as a matter of fact, we do not quite agree about anything whatsoever! Therefore, we may at any rate claim still to be very professional to the full extent of the proverb, that "doctors differ."

And as to whether we should speak of the idea of similars as a law or as a rule, the contention that it is a rule rather than a law is, I submit, quite groundless. But as some have been captivated by the reasonings of those who pose as the champions of rule as against law, it might not be amiss to point out that the whole contention for the rule is based upon the poor grammar of the disputers. I have, thus far, never known of a German or a Frenchman go in for "rule," and that for the very sufficient reason that they understand the use of the subjunctive mood, which cannot be said of all Britishers, no matter how learned they may be. In order to really understand Hahnemann on this point, it is absolutely essential that one understand Latin and German composition, more particularly in regard to the subjunctive. Those who contend for "rule" had better scuttle out of their position as quietly as they can, lest some one, some of these fine days, take the trouble to pour out a vial of wholesome ridicule upon their "rule." The same remarks apply in regard to the question of the noted formula of the homœopathic school—viz., whether should we say *similia similibus curantur*, or *similia similibus curentur?* Of course, the reply is that both are correct, they both express precisely the same thing, only one is in the indicative and the other in the subjunctive. I do not admit that it is in the imperative. In some of the old Hermetic works you will find it put *similia similibus curari*, which is, of course, precisely the same thing, only in another mood. You will also find *simile a simila curari;* hence, it is really, in more ways than one, merely a matter of mood.

However, everything in this world is comparative, and, comparatively speaking, we do agree that like cures like ; and be it notion, principle, law, rule, or method, we so far agree to admit that these words, *similia similibus curantur*, express something positively demonstrable in clinical life. All this falls within that phase of the development of Homœopathy anterior to the sojourn of Hahnemann at Coethen. And this part has been really almost completely exhausted, so let us go over to Coethen and hear the oracular pronouncement that all chronic disease is primarily due to three somethings—*psora*, *syphilis*, and *sycosis*.

When a man comes out of the land of darkness of school teachings, and throws over school physic (I do not mean brimstone and treacle, which was *my* school physic), and passes into the comparative glare of Hahnemannic therapeutics, he is generally considered perturbated by the violent change of climate—*i. e.*, from darkness to light. He requires some time to acclimatize. At first he usually has an acute attack of homœopathic enthusiasm, a veritable fever that yields neither to Aconite nor to Pyrogen, and he makes a *tabula rasa* of everything, and a good deal besides.

But when a few failures have sobered him down a wee, he goes back into himself, and finds out a few things for himself.

He finds that *Belladonna* will cure the delirium of tuberculosis of the meninges, and other of its symptoms, but the patient in the end dies all the same. He gives *Baptisia*, *Arsenicum*, serpent poisons, acids, etc., in low fevers, but his patients are very apt to die in the end, all the same. He has a patient given to picking his nose, or things in general, and after considering the merits of *Arum triphyllum*, *Conium*, *Helleborus*, *Lachesis*, *Selenium*, *Stramonium*, and the like, and exhibiting them, he finds—the worms live on still!

In fact, he learns to discriminate, and to differentiate between true initial and all-along-the-line similarity, and that which is ultimate and superficial only. When a man in his homœpathics arrives at this stage of his developmental process, he is apt to do one of three things, viz.: he may, 1st, throw your Homœopathy clean overboard; or, 2d, admit the limitedness of its sphere of application; or, lastly, he may set about procuring a pathology to fit his therapeutic doctrine. I have gone through all these stages myself now, and am beginning to understand the Coethen ætiologic phase of Homœopathy. If space would allow, I would seek to encompass this ætiologic phase of Homœopathy in its entirety; but, as it will not permit of this, I have chosen only one of the three Hahnemannic, chronic, so-called miasms for consideration, and that sycosis.

I have a special reason for choosing sycosis. I mean the sycosis of Hahnemann, and not the *sycosis autorum*, viz., our knowledge on the subject has much increased of late years, for science has been shining upon it.

Now, leaving syphilis and psora quite out of consideration, I propose to inquire into the Hahnemannic doctrine of sycosis in the light of modern science and experience.

First of all, I would make a preliminary observation in respect of the word *miasm*, which is current in homœopathic literature in a very peculiar sense. Hahnemann himself calls the supposed causes of chronic diseases *miasms*, and his translators carefully and conscientiously translate the word by itself!

Now, in English *miasm* means an infection floating in the air; the effluvia or fine particles of any putrifying or noxious bodies rising and floating in the atmosphere—in fact, exhalations. Therefore it is hardly accurate to use the English word *miasm*, or its pure Greek form *miasma*, as the English equivalent of the word "miasma" as used by Hahnemann, or if you do, you must carefully define the use of the word first, for our word miasm, being derived from μίαινω to soil, to defile, to pollute, to dirty, might etymologically stand as the translators of Hahnemann have it, but τὸ μίασμα means not only a defilement, a soiling, a befouling, but also an impure exhalation, in which restricted sense only it has come into use in English. Miasm in our vernacular means impure particles or effluvia in the atmosphere, and nothing else. What Hahnemann meant when he used the Germanized miasma was not at all what we understand by miasm, but was rather what we now understand by *virus* when applied to the primary form of a disease, and *taint* when used to denote the latter phases. If in speaking English in these days we talk of the syphilitic virus or taint, the gonorrhœal virus or taint, the virus of itch, the itch-taint, we are expressing ourselves, so far as the words are concerned, accurately, and everybody knows what we mean, but when we speak of the miasms of these diseases we are really, as I must submit, using jargon, and so gratuitously mystifying ourselves. Ague is supposedly due to a miasm, syphilis to a virus. So much, therefore, for the word miasm, as wrongly used in homœopathic literature. I say wrongly, because it tends to obscure, and in all conscience the thing is obscure enough without any verbal mystifications.

Now, let us go on to inquire what Hahnemann understood by sycosis. The highest English authority on the exegetics of Homœopathy is, I think all will admit, Dr. Dudgeon, and he says (*Lectures on the Theory and Practice of Homœopathy*, 1854, p. 300): "As regards the third of Hahnemann's chronic miasms sycosis, or the condylomatous venereal disease, the notion of its independent nature has been considerably contested, not alone by allopaths, but also by some of our own school. The disease always arises in consequence of impure coitus, and appears in the form of dry or nasty-looking, or soft and spongy excrescences in the form of a cockscomb or cauliflower, easily bleeding, and secreting a fœtid fluid, and sometimes accompanied by a sort of blennorrhœa from the urethra. Their seat is the glans or foreskin in the male, the vulva and its appendages in the female. Their removal by the ligature or cautery, actual or potential, is, according to Hahnemann, followed by similar growths on other parts of the body or other ailments, the only one he mentions being shortening of the flexor tendons, particularly of the fingers.

"It is, Hahnemann alleges, the rarest of the three chronic miasms, and, as I before observed, it is very doubtful if it be a peculiar disease, and not rather a form of syphilis. The secondary effects Hahnemann describes as arising from it must certainly be rare, for I can state from my own experience that I know several persons who have had such venereal condylomata burnt off many years ago, and who have never had the slightest trace of those after-effects Hahnemann alludes to; though at the same time I am bound to admit that I think I have observed a connection of certain pseudo-rheumatic affections and inveterate gleets with the fig-wart disease." Thus far Dr. Dudgeon.

So the *only* after-effect of the fig-wart disease mentioned by Hahnemann is a shortening of the flexor tendons, particularly of the fingers, and yet Dr. Dudgeon speaks of "those after-effects Hahnemann alludes to!"

It can thus hardly be maintained that Dr. Dudgeon puts sycosis before us in a very clear light, though his remarks in regard to gonorrhœal rheumatism shows the accurate observer, and John Hunter had observed the same thing long ago. That people do get venereal warts admits of no doubt whatever, that they are a form of syphilis, as stated by Dudgeon, is not now generally admitted.

Hahnemann very clearly differentiated between syphilis and sycosis, because he found *Mercurius* helped to cure syphilis but not fig-warts, and modern experience and science are seemingly on Hahnemann's side on this point. Dudgeon very properly objects to consider diseases as sycotic simply because they can be curatively modified by *Nitric acid* and *Thuja*. But then we cannot entirely ignore the aid obtainable from this source; for instance, a very bad chronic ulcerated sore throat that yields straightway to full doses of the *Iodide of Potassium* tells a tale we all understand without any commentator. I have long been puzzled with Hahnemann's divisions of drugs—*i. e.*, how he arrived at them—and I am beginning to suspect that he made them largely by an appreciation of the *ex juvantibus et nocentibus* teachings. And a number of his indications are, beyond any doubt, derived from the time-old *signaturæ rerum naturalium*. *Thuja* to wit.

Now, I complain that the great exegete of Homœopathy, Dr. Dudgeon, whom we all delight to honor, devotes too little attention to the doctrine of sycosis; he neither establishes it nor does he demolish it. Dr. Dudgeon mentions it in passing, throws doubt upon it, and then leaves it. Dudgeon's doubt as to the separate nature of the condylomatous venereal disease is based upon his observations that he had known persons in whom the condylomata were burnt off many years ago, and yet the flexor tendons of their fingers had never become shortened! I can say the same, and, no doubt, we all can, but we have equally seen plenty of people who had syphilis many years ago, and who have never had any later manifestations of the disease, but that in no way militates against the specific nature of late, later, and latest manifestations of syphilis where they do occur.

Dungeon speaks with no great respect of those homœopathic practitioners who have regarded ordinary warts as evidence of sycotic infection, because Hahnemann distinctly declares such warts as of psoric origin. This looks like a formidable indictment, but one which vanishes when more closely examined. It is quite true that Hahnemann puts common warts, encysted and other tumors, down to the very large account of psora, but he does not say "all" warts, only some. And herein lies *des Pudels Kern*, as I will proceed to show.

Let us now go to Hahnemann's own account of sycosis and see if it tallies with Dudgeon's. Turning up the *Chronische Krankheiten* we come upon the chapter devoted to the subject, and find it is just as scant and unsatisfactory as Dudgeon's exegesis of it. Hahnemann only devotes one small chapter of four pages to it, and Dudgeon's account of it is quite correct, except that he fails to point out the strange statement by Hahnemann that sycosis is an epidemic affection, "*Nur von Zeit zu Zeit herrschend war*," and ever getting more and more rare.

Common gonorrhœa, Hahnemann says, does not appear to penetrate the whole organism, but only to irritate the urinary organs locally.

His remedies for sycosis are a few globules of *Thuja*$^{30}$ and *Nitric acid*$^{30}$. His remedies for the common clap are a drop of fresh parsley juice, if there is much urging to urinate, and *Copaiva balsam;* about one drop of the mother tincture when there is less inflammation, and if these do not do the trick, why you get a gleet which is *psoric.*

According to Hahnemann, therefore, there are two kinds of gonorrhœa, or clap; the one with condylomata, which is constitution infecting, and in which the urethral flux may occasionally but not often be wanting, and which constitutes his sycosis, and which must be monoposically cured by *Thuja*$^{30}$ and *Acid nit.*$^{30}$, leaving each from twenty to forty days' time of action.

I would here remark, with some emphasis, that Hahnemann very distinctly differentiates between local irritation and an organismic evil in regard to the dose; when he wants to treat the organ or the part, *topico*—specifically he uses the mother tincture—or simple juice of the plant—and when he wants to treat the organism he uses the higher dilutions; and I may say that my own observations tally with this view exactly, with this difference, viz., that for the topic action the small material dose has to be often repeated. Before we go any further, let us note that Hahnemann uses the word *miasm* for the cause of the common non-condylomatous clap as well as for the other.

Let us now resume for a moment. According to Hahnemann there are two kinds of clap, the condylomatous, which is constitutional, and is to be cured monoposically by *Thuja* and *Nitric acid;* and the common clap, which is a merely local affection of the urethra, and is to be cured by the juice of *Petroselinum sativum,* monoposically also, if much urging to urinate; or a drop of the alcoholic solution of the *Balsam of Copaiva* when there is less inflammatory irritation.

This is, practically, all that Hahnemann tells us about his sycosis and his common gonorrhœa.

We have now considered Dudgeon as exegete and Hahnemann as the originator of the doctrine of sycosis, but we have herewith not overmuch light, and conceptions not too clear. During the past forty years there have been very numerous authors who have written on Hahnemann's sycosis. Bœnninghausen, Wolf, Grauvogl, Hering, H. Goullon, and many others, and it would be very interesting to follow these thinkers in their yearnings and gropings after truth, in their desire to harmonize the facts of science with their veneration of the master.

But I am afraid the task is too great, and, moreover, I prefer another plan. I suggest that we take, first of all, Hahnemann himself, as likely to know most of his own mental offspring. I suppose the majority of us feel that we know most of our own children after the flesh, and a man may fairly, I should think, be considered an authority on his own mental offspring also.

I quite agree with the principal exegetists of Hahnemann that it does not follow that because *Thuja* and *Nitric acid* may cure a complaint that therefore said complaint is of a sycotic nature, as Hahnemann understands it; but, inasmuch as we conclude that grave ulcerations, which readily yield (at least temporarily) to the *Iodide of Potassium,* are in all probability of a certain specific nature, so in like manner it may fairly be conceded, at least for the sake of study and argument, that what can be cured by the two grand antisycotics may very probably be of a sycotic nature.

Let us take merely the standpoint of probability, that much may be safely conceded without any great danger to scientific truth. Therefore, I invite you to consult Hahnemann on the subject of sycosis under the headings of *Thuja* and *Nitric acid.*

Well, the Hahnemannian pathogenesis of *Thuja* does not help us a bit, and, oddly enough, *Nitric acid* is classified by Hahnemann as what? as an antipsoric! So we see that Hahnemann classifies *Nitric acid* as an antipsoric after having mentioned it as second in order for the radical cure of sycosis. Then, again, although he classifies *Nitric acid* as an antipsoric, he mentions warts (of the psoric kind?) and also condylomata and inguinal adenomata as curable by *Nitric acid*, while the symptomatology of this acid clearly portrays gonorrhœa (S. 375 to 389).

Hughes tells us that our only pathogenesis of Nitric acid was first published in the second edition of the *Chronic Diseases*, containing 1,426 symptoms. This cannot be correct, for my edition is the first, 1828, and it contains a pathogenesis of Nitric acid, with 803 symptoms.

Well, with all this we get no clear conception of Hahnemann's sycosis, as an adequate basis for the huge structure which some of his disciples have built upon it, and which is the sycosis of the homœopathic authors, but I am not satisfied that it is Hahnemann's.

I propose now to consult Ameke's *History of Homœopathy* on the point, and on page 138 of Drysdale's Translation, read "Besides this 'psora' there were other fundamental causes, viz., 'sycosis,' the phenomena connected with gonorrhœa and 'syphilis.' Though there may have been some substratum of truth in these views, Hahnemann nevertheless far transcended the limits of probability, and fell into a great error." Here, then, according to Ameke, as translated by Dr. Alfred Drysdale, and edited by Dr. Dudgeon, we find sycosis defined as "the phenomena connected with gonorrhœa." So, according to this, sycosis and the clap disease, the *Tripperseuche* are identical. This positive statement of the identity of the gonorrhœal disease in its entirety and the sycosis of Hahnemann so surprised me that I turned to the original and find the translator has interpolated the definite article *the*, which makes all the difference. Ameke's words are "ausser dieser Psora blieben noch als Grundursachen übrig die Sycosis, mit dem Tripper zusammen-hängende Erscheinungen, und Syphilis," and these mean "sycosis, phenomena connected with clap," not *the* phenomena.

The words of Ameke, viz., "there may have been some substratum of truth in these views" (of Hahnemann) really pretty nearly epitomize the actual attitude of the homœopathic practitioners of the world at large. Speaking broadly, you to whom these words are addressed do *not* accept the ætiologic phase of Homœopathy, and yet almost every man of you is daily, almost hourly, influenced by it in his modes of thought, of practice, and of writing and speaking. You do not accept the doctrines of psora, syphilis, and sycosis, and yet you do not quite reject them; you seem to think there is something in them after all.

Now, to keep within the bounds of my plan, viz., of sycosis, surely we ought to be able to *know* whether the doctrine of sycosis is true or false. Indeed, I think it about time sycosis were elevated from the position of a scholastic doctrine to that of positive scientific demonstration, at least clinically, or else cast out altogether; for it must be manifest that there either is, or there is not, a condylomatous venereal disease which we call sycosis.

At this stage of our inquiry we are encountered with a difficulty, for to my mind it is very questionable whether sycosis and the entire gonorrhœal disease are identical. We have seen that Hahnemann differentiates two kinds of clap, the one a local affection of the urinary organs, and the other sycosis, in which there may be no urethral pyorrhœa or blennorrhœa at all. And this quite coincides with what we no doubt have all seen over and over again, viz., condylomata, or *verrucæ accuminatæ*, in persons who have had no gonorrhœa at any time; but in all the cases which I have ever observed, impure coition had probably taken place (the hereditary ones in children always excepted), and hence these warts are certainly venereal; but are they always gonorrhœal? To say that the principal exegetes of Homœopathy and the pro-sycosis writers, such as H. Goullon, and the various and numerous authors quoted by him in his admirable prize essay on *Thuja and the Lues Gonorrhoica*, accepts sycosis as synonymous with the whole gonorrhœal disease, which Autenrieth and other writers before and at the time of Hahnemann fully recognized and proclaimed as due to a constitution-infecting virus, and which they termed *Tripperseuche*, or clap disease, and which they also ascribed to a miasma or virus, as did Hahnemann. To say this does not satisfy my mind that Hahnemann thought the gonorrhœal virus the primary cause of fig-warts and other constitutional ailments. I think everything must hinge upon the answer to this question. I have weighed the matter carefully, and have come to the conclusion that sycosis for Hahnemann was the *condylomatous venereal disease* indeed, and nothing else, and *not* the *Tripperseuche*, or clap-disease, of Autenrieth in its entirety.

If you will take the trouble to read the greater medical writers of Germany of the first four decades of this century, you will find (and I am sure Drysdale, Dudgeon, Hughes, H. Goullon, to name no others, will all agree with me) that gonorrhœa was considered by very many of them as a *Seuche*, or constitutional affection, and as the prime cause of many specifically gonorrhœal ailments or manifestations, only one of which is the condyloma.

The clap disease, *die Tripperseuche,* was a recognized prime cause of chronic disease years before our founder promulgated his sycosis, and if you admit that sycosis and clap-disease are synonymous terms, then sycosis is not the mental property of Hahnemann at all; this much is certain, either sycosis and clap-disease are not the same thing, or else if they are, there is no such a thing as sycosis to be attributed to the genius of the founder of Homœopathy.

We must not forget that Hahnemann differentiates two kinds of clap, the common variety and that of the condyloma, so he evidently did not include the whole clap disease in his sycosis.

It is seemingly no use for us to hunt about in Hahnemann's works for any real enlightenment on the subject of sycosis, as they contain none; and why? Simply because Hahnemann himself had but very little knowledge on the subject, as he practically admits on page 63, of vol. I, of his *Chronische Krankheiten.* I should not be surprised if he had set aside sycosis for study and consideration in a future time, but apparently that time never came—that is, it never came so far as we know; possibly the Paris MSS. may contain something on the subject.

We are then brought face to face with this primary question, Is the sycosis of Hahnemann identical with the gonorrhœal disease of Autenrieth? If so, then it is not the property of Hahnemann; and if not identical, what is it? syphilitic, gonorrhœal, chancroidal, or a separate and independent disease *sui generis?*

These points being settled, we could proceed to a comparison of gonorrhœa in its constitutional aspects, with the sycosis delineated in the original works of Hahnemann. For I for one cannot admit that the *sycosis autorum homœopathicorum* is the sycosis as painted by Hahnemann himself.

NOTE.—The above quoted paper of Dr. Burnett's was prepared by him as an introductory to an extended study of gonorrhœa and sycosis; the more practical consideration of the subject was prevented by sickness in his family. It is to be hoped Dr. Burnett will complete his essay at an early day, for all true homœopaths are interested in this subject, and would enjoy reading an essay upon it from the pen of such an interesting writer as the Doctor.—EDITORS H. P.

